# The carbohydrate-insulin model does not explain the impact of varying dietary macronutrients on the body weight and adiposity of mice

**DOI:** 10.1016/j.molmet.2019.11.010

**Published:** 2019-11-16

**Authors:** Sumei Hu, Lu Wang, Jacques Togo, Dengbao Yang, Yanchao Xu, Yingga Wu, Alex Douglas, John R. Speakman

**Affiliations:** 1 State Key Laboratory of Molecular Developmental Biology, Institute of Genetics and Developmental Biology, Chinese Academy of Sciences, Beijing, 100101, PR China; 2 University of Chinese Academy of Sciences, Shijingshan District, Beijing, 100049, PR China; 3 Institute of Biological and Environmental Sciences, University of Aberdeen, Aberdeen, AB24 2TZ, Scotland, UK; 4 CAS Center for Excellence in Animal Evolution and Genetics (CCEAEG), Kunming, PR China

**Keywords:** Carbohydrate-insulin model (CIM), Energy intake, Energy expenditure, Insulin, Mice

## Abstract

**Objectives:**

The carbohydrate-insulin model (CIM) predicts that increases in fasting and post-prandial insulin in response to dietary carbohydrates stimulate energy intake and lower energy expenditures, leading to positive energy balance and weight gain. The objective of the present study was to directly test the CIM's predictions using C57BL/6 mice.

**Methods:**

Diets were designed by altering dietary carbohydrates with either fixed protein or fat content and were fed to C57BL/6 mice acutely or chronically for 12 weeks. The body weight, body composition, food intake, and energy expenditures of the mice were measured. Their fasting and post-prandial glucose and insulin levels were also measured. RNA-seq was performed on RNA from the hypothalamus and subcutaneous white adipose tissue. Pathway analysis was conducted using IPA.

**Results:**

Only the post-prandial insulin and fasting glucose levels followed the CIM's predictions. The lipolysis and leptin signaling pathways in the sWAT were inhibited in relation to the elevated fasting insulin, supporting the CIM's predicted impact of high insulin. However, because higher fasting insulin was unrelated to carbohydrate intake, the overall pattern did not support the model. Moreover, the hypothalamic hunger pathways were inhibited in relation to the increased fasting insulin, and the energy intake was not increased. The browning pathway in the sWAT was inhibited at higher insulin levels, but the daily energy expenditure was not altered.

**Conclusions:**

Two of the predictions were partially supported (and hence also partially not supported) and the other three predictions were not supported. We conclude that the CIM does not explain the impact of dietary macronutrients on adiposity in mice.

## Highlights

• Higher fasting insulin related to inhibited lipolysis and leptin pathways in sWAT, supporting CIM.
• Higher fasting insulin related to inhibited hypothalamic hunger pathway, contrasting CIM.
• Fasting insulin decreased with higher dietary carbohydrate, overall contrasting CIM.
• Higher dietary carbohydrate did not lead to greater EI/adiposity, or lowered EE.

## 1 Introduction

Obesity is widely accepted to be a problem of energy imbalance [1]. Individuals with obesity must have spent a substantial period of time in positive energy balance. However, while this is a necessary pre-condition, this energy balance model provides little explanation because it does not elucidate why the original positive energy balance occurred. Historically, a suggested major factor driving obesity was lowered energy expenditure due to the increasingly sedentary modern lifestyle [2]. However, more recent analyses have suggested this interpretation is untenable [3] and that the major driver of the epidemic is more likely overconsumption of energy.

Several different models have been proposed to explain why some individuals over-consume calories. The carbohydrate-insulin model (CIM) suggests that high levels of dietary carbohydrates elevate insulin secretion, which suppresses fatty acid oxidation and the release of fatty acids from adipose tissue while promoting lipogenesis [4,5]. This creates a state of cellular “internal starvation” that drives both increased food intake and decreased energy expenditure [[4], [5], [6]], and thus obesity [7]. This model led to the corresponding solution to the obesity crisis that individuals should cut out dietary carbohydrates and instead favor fat and protein in their diets via “low carbohydrate, high fat” diets, which have been the subject of several popular books [[8], [9], [10], [11]]. However, this dietary approach remains controversial [12,13], particularly because it can conflict with current dietary guidelines to reduce saturated fat consumption [14].

Several studies have tested the effectiveness of low carbohydrate, high fat (LCHF) diets and like most diet studies have shown variable impacts on body weight management [[15], [16], [17], [18], [19], [20], [21], [22], [23]]. There are potentially many reasons underpinning these different outcomes. However, a major issue is the difficulty of performing long-term diet studies of humans where precise control over dietary intakes is difficult. In a recent study, for example, in which subjects were provided free food for 6 months, there was still substantial intake of extra food outside the diet prescription [23]. The objective of the present study was to directly test CIM predictions using the mouse as a more tractable model system, where diets can be more easily manipulated and intake can be measured much more accurately than in humans [24]. The CIM makes a number of specific predictions that we tested: A) The fasting and post-prandial insulin levels will increase in relation to the dietary level of carbohydrates, and the fasting glucose will decrease reflecting the “starvation state.” B) Elevated insulin leads to changes in fat tissue metabolism favoring deposition, including elevated levels of lipogenesis and reduced lipolysis, directly related to the circulating insulin levels and dietary carbohydrate content. A state of net de novo lipogenesis would be detected by a shift in the respiratory exchange ratio (RER) to exceed the expectation from the food quotient (FQ). C) Increasing dietary carbohydrate content leads to greater food intake and lowered energy expenditure. D) The insulin-generated state of cellular starvation stimulates brain pathways regulating food intake and inhibits peripheral pathways leading to reduced expenditure such as reduced adipose tissue browning. E) The resultant positive energy balance leads to greater adiposity and overall increases in body weight.

## 2 Materials and methods

To test the CIM's specific predictions, we conducted two studies.

1) The acute post-prandial insulin measures in relation to dietary CHO intake
2) The chronic measurements of body weight, body adiposity, energy expenditure, fasting glucose and insulin, and other related measurements

### 2.1 Ethical statement and registration

All procedures were approved by the Institutional Review Board, Institute of Genetics and Developmental Biology, Chinese Academy of Sciences, approval numbers AP2014011 and AP2019027. This project was also registered with the Open Science Framework (https://doi.org/10.17605/OSF.IO/YH9GZ).

### 2.2 Mice

Male C57BL/6 mice were used. All mice were purchased at age 8 weeks from Charles River and acclimated to an animal house for 2 weeks. For experiment one, 35 C57BL/6 mice were used to investigate the effect of carbohydrate content on post-prandial insulin secretion.

For experiment two, a total of 480 male C57BL/6 mice were used to investigate the effect of carbohydrate content on adiposity, at 20 mice per diet. We exposed these mice to 4 series of 6 different diets that varied in the carbohydrate content and fixed protein content (details given below). Based on the previously reported variations in the response of C57BL/6 mice to a high fat diet [25], a power analysis suggested a sample of 20 per group was necessary to detect an effect size of 0.3 g/day in the mean food intake between groups with 80% power at alpha = 0.05 in a one-way ANOVA with 6 levels.

All mice were housed individually in a specific pathogen-free facility (environmentally controlled: temperature 22–24 °C, 12:12 LD cycle, with lights on at 7:30 am, following previous suggestions [26]). They had ad libitum access to food and water and were monitored for health status daily over the entire experimental period. All mice were fed a standard diet with 10% fat and 20% protein (D12450B, Research Diets Ltd) for 2 weeks for baseline adjustment, followed by random allocation to different diet groups. After 12 weeks on the experimental diets, all mice were euthanized with carbon dioxide and then dissected.

### 2.3 Experimental diets

In total, the mice were fed 4 series of diets consisting of 6 different diets per series (total = 24 diets). Full details of these diets are shown in Table S3. This version updates the previous table [27]. We fixed the level of fat at 60% (codes: D14071601-D14071606) or 20% (codes: D14071607-D140716012), or the level of protein at 10% (codes: D14071602, D14071608, D14071613-D14071618) or 25% (codes: D14071605, D14071611, D14071619-D14071624) by energy and then varied carbohydrate content. The protein source, fat source, and other related diet details can be found in Table S3 or at the Research Diets website (https://www.researchdiets.com/blog/posts/macronutrientdiets).

### 2.4 Study one

For acute post-prandial insulin measurement, 8-week-old male C57BL/6 mice were fed 7 diets with variable carbohydrate content. A total of 35 mice were used with 5 mice per diet. All mice were fasted from 9 am to 6 pm and then fed different diets. Following 90–120 min of receiving diets, the mice were sacrificed for the collection of post-prandial blood samples, and the amount of food they had eaten was weighed. The blood samples were maintained at room temperature to clot for 1 h and then centrifuged for 30 min at 3500 rpm. The resultant serum samples were then collected to measure the post-prandial insulin.

The serum insulin levels were determined using an Ultra-Sensitive Mouse Insulin ELISA Kit with a wide range assay (90080, Crystal Chem Inc., Downers Grove, IL, USA) following the manufacturer's instructions. The kit is based on a sandwich enzyme immunoassay. On an antibody-coated microplate, 95 μL of diluent and 5 μL of sample (or insulin standard) were mixed and then incubated for 2 h at 4 °C to enable the insulin in the sample to bind to guinea pig anti-insulin antibody. After washing off the unattached samples, 100 μL of anti-insulin enzyme conjugate was added to the wells. They were then incubated for 30 min at room temperature to bind the horseradish peroxidase (POD)-conjugated anti-insulin antibody to the guinea pig anti-insulin antibody-mouse insulin complex. The wells were then washed again and 100 μL of enzyme substrate solution was added and reacted for 40 min at room temperature in the dark for the detection of POD conjugate with 3,3′,5,5′-tetramethylbenzidine (TMB) substrate. The reaction was then stopped by adding 100 μL of stopping solution. The absorbance was read using a plate reader (A_450_ and A_630_ values). Standard curves were generated by plotting the absorbance vs the corresponding concentration of mouse insulin standard to calculate each plate.

### 2.5 Study two

#### 2.5.1 Food intake, body mass, and body composition measurement

Following the 2-week baseline period, all of the mice were exposed to the experimental diets for 12 weeks. Their body weight (BW) and food intake (FI) were measured daily throughout the experiment. The body composition (mainly fat mass and lean mass) of all mice were measured weekly using an EchoMRI Body Composition Analyzer [28] with canola oil as the standard. Their food intake was measured by the food weight on the first day subtracting the food weight on the second day from the food hopper and was eliminated if a problem was ascertained [27].

#### 2.5.2 Physical activity and energy expenditure measurement

The mice were transferred to a TSE PhenoMaster/LabMaster system for 2 days after 10 weeks of diet switching, which was sufficient for an accurate measurement of their energy expenditure [29]. Their oxygen (O_2_) consumption (mL/min), carbon dioxide (CO_2_) production (mL/min), locomotor activity (counts), and food intake (g) were recorded as reported [27].

The respiratory exchange ratio (RER) was calculated using the CO_2_ production divided by the O_2_ consumption (VCO_2_/VO_2_). The energy expenditure (EE) was calculated according to the Weir equation: EE (kJ/day) = ((3.9 x VO_2_ (mL/min) + 1.1 x VCO_2_ (mL/min)) x 1440 (min)/1000 × 4.184 [30]. The daily energy expenditure (DEE) was calculated using the average VO_2_ and VCO_2_ over the measurement period, and the resting energy expenditure (REE) was calculated using the minimum VO_2_ and VCO_2_ over the measurement period. We also calculated the energy expenditure over the entire 12-week study period from the data on the body weight, body fat mass, and food intake changes using a program provided by Guo and Hall (2009) [31] and Guo et al. (2009) [32].

The food quotient (FQ) was calculated for all of the diets. The FQ for the carbohydrate, fat, and protein were 1.0, 0.7, and 0.8, respectively [33]. The FQ equation used in the study was FQ = 1.0 x carbohydrate (%) + 0.7 x fat (%) + 0.8 x protein (%) [34]. The discrepancy between the RER and the FQ was then calculated by subtracting the FQ from the RER.

#### 2.5.3 Fasting blood glucose and insulin measurement

At week 10, the mice were fasted for 4 h. Blood samples were then collected from their tail veins and the glucose levels were measured using a glucometer. After 12 weeks, the mice were sacrificed and their fasting blood samples were collected and maintained at room temperature to clot for 1 h. Following clotting, the blood samples were centrifuged for 30 min at 3500 rpm and the upper serum layers were then separated to measure the fasting insulin concentration.

#### 2.5.4 RNA extraction and transcriptome analysis

The hypothalamic and subcutaneous white adipose tissue (sWAT) samples were collected and pooled samples were then obtained as previously described [27]. The total RNA in the hypothalamic and sWAT samples was extracted using an RNeasy Mini Kit (74104, Qiagen) according to the manufacturer's protocols. The sequencing of all RNA samples was performed using an Illumina NextSeq 500 sequencer via 75 bp reads from the paired ends. The paired-end reads were mapped to the Mus musculus genome (GRCm38) and the mapped reads were then counted against the GTF file of GRCm38 from Ensembl (release 83). The genes with counts per million (CPM) ≥ 1 in at least one diet group were retained [35].

### 2.6 Statistical analysis

Statistical analyses were conducted using Microsoft Excel, IBM SPSS 20, and the R platform [36]. All values are expressed as mean ± SD. Whole animal oxygen consumption and energy expenditure were evaluated using one-way ANOVA with Bonferroni's post hoc test. Differences were considered significant if p < 0.05. Pearson's correlation between the transcriptome data counts and the fasting insulin levels were calculated using the Hmisc package (version 4.1.1, R version 3.5.1). Pearson's correlation coefficients and p-values were loaded into the Ingenuity Pathway Analysis (IPA) program for core analysis (Ingenuity Systems, https://www.qiagenbioinformatics.com/products/ingenuity-pathway-analysis/). The p-value of the overlap between our dataset and a particular biological attribute was calculated using the right-tailed Fisher's exact test via the IPA. The Benjamini–Hochberg correlation for multiple testing of canonical pathways was conducted using the IPA. We visualized the hunger signaling pathway in the hypothalamus, the browning, insulin, and leptin signaling pathways in the sWAT using custom–built pathways [37], and the canonical lipolysis pathways via the IPA.

## 3 Results

### 3.1 Prediction A): the post-prandial insulin levels increased in relation to the increasing carbohydrate intake but the post-prandial glucose was unchanged. The fasting glucose and insulin levels both decreased in relation to the dietary carbohydrate intake

**Figure 1:**
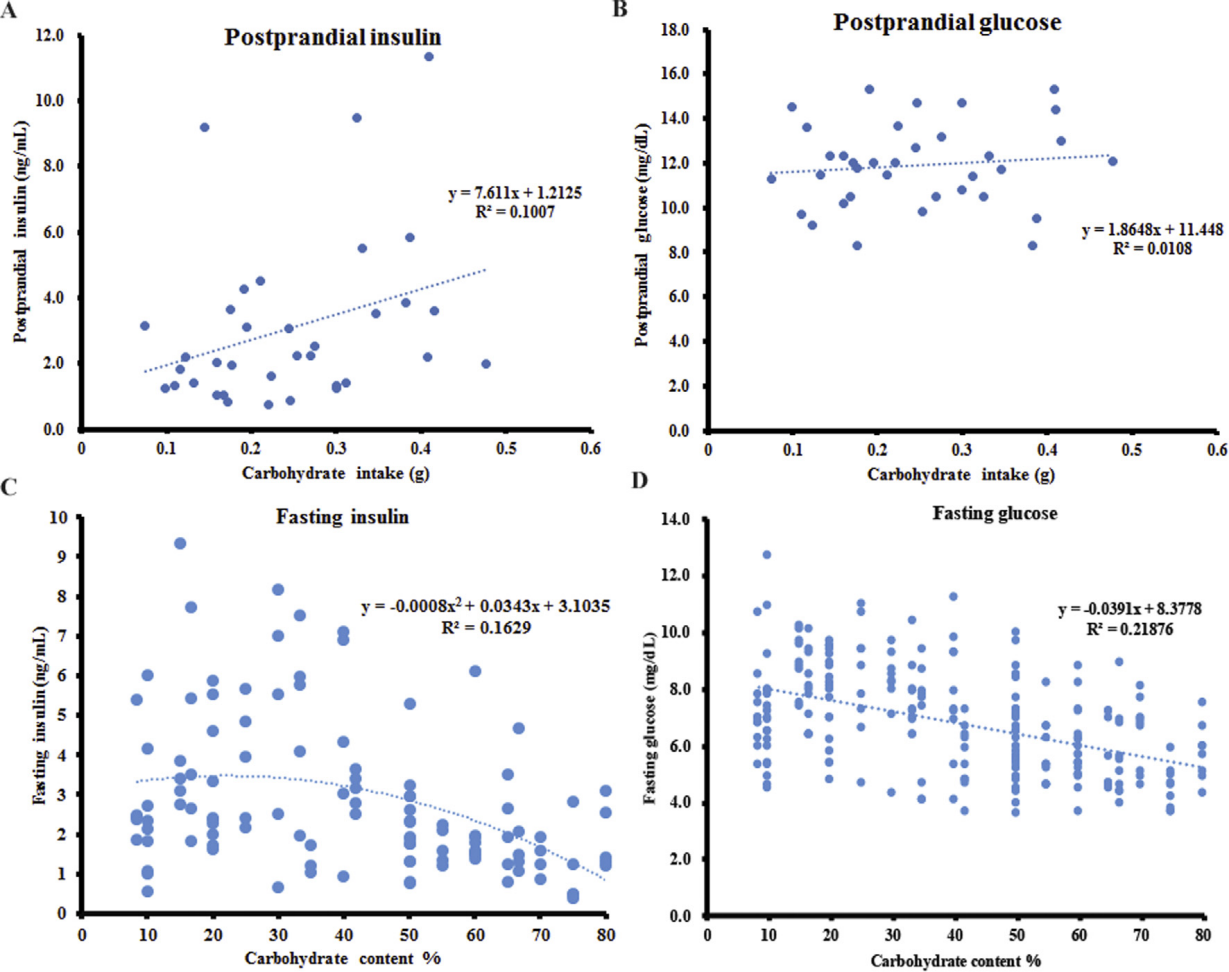
The insulin and glucose levels of the mice in both post-prandial and fasting states. The values were the measured values of each individual. The post-prandial (A) insulin and (B) glucose levels of the mice 90–120 min after feeding. A total of 35 mice were used, with 5 mice per diet. The fasting (C) insulin and (D) glucose levels of the mice following 12 weeks on experimental diets. A total of 240 mice were used, with 10 mice per diet. See also Figure S1.

The post-prandial insulin and glucose levels of the mice were measured after 90–120 mins following acute exposure to diets with variable carbohydrate content (from 16.7% to 80%). There was a positive relationship between the post-prandial insulin levels and carbohydrate intake, which almost reached significance (linear regression: F_1,33_ = 3.701, R^2^ = 0.101, p = 0.063) (Figure 1A). No significant relationship was observed between the post-prandial insulin levels and fat intake (linear regression: F_1,33_ = 0.79, R^2^ = 0.023, p = 0.381) or protein intake (linear regression: F_1,33_ = 0.39, R^2^ = 0.012, p = 0.539). There was no significant relationship between the post-prandial glucose levels (90–120 mins after food intake) and carbohydrate intake (linear regression: F_1,33_ = 5.023, R^2^ = 0.011, p = 0.552) (Figure 1B). A generalized linear model with the post-prandial glucose levels as the dependent variables and the carbohydrate, protein, and fat intake as predictors showed that all three macronutrients were not significantly associated with the post-prandial glucose levels 90–120 mins after food intake (p = 0.954 for carbohydrate, p = 0.455 for protein, and p = 0.088 for fat intake).

In a separate experiment, fasting blood samples were collected to measure the fasting insulin levels following a 12-week exposure to diets with varying carbohydrate content. There was a significant negative relationship between the fasting insulin levels and dietary carbohydrate content, when the carbohydrate content increased from 8.4% to 80% (polynomial regression: F_2,113_ = 10.99, R^2^ = 0.163, p = 4.34 × 10^−5^) (Figure 1C). The fasting blood glucose of the mice following 10 weeks on diets with variable carbohydrate content decreased in relation to the increasing dietary carbohydrate content (linear regression: F_1, 228_ = 63.84, R^2^ = 0.219, p = 6.66 × 10^−14^) (Figure 1D). When the fasting insulin levels or glucose levels were normalized to the body fat mass, neither the fasting insulin levels (linear regression: F_1, 114_ = 4.09 × 10^−4^, R^2^ = 3.59 × 10^−4^, p = 0.984) nor the glucose levels (linear regression: F_1, 228_ = 3.37, R^2^ = 0.015, p = 0.068) were significantly related to the dietary carbohydrate content.

### 3.2 Prediction B): increased fasting insulin was correlated with the inhibited lipolysis, leptin, and the insulin signaling pathways, although the lipogenesis pathway was not altered

To investigate the association between the fasting insulin levels and fat tissue metabolism, we estimated Pearson's correlations between the fasting insulin levels and the global tissue gene expression levels in the subcutaneous white adipose tissue (sWAT) using RNA sequencing (RNA-seq). This was performed on the RNA extracted from the sWAT of the mice exposed to the experimental diets for 12 weeks. There were 2863 genes significantly correlated with the fasting insulin levels, with 1128 genes positively correlated and the remainder negatively correlated (Table S1).

**Figure 2:**
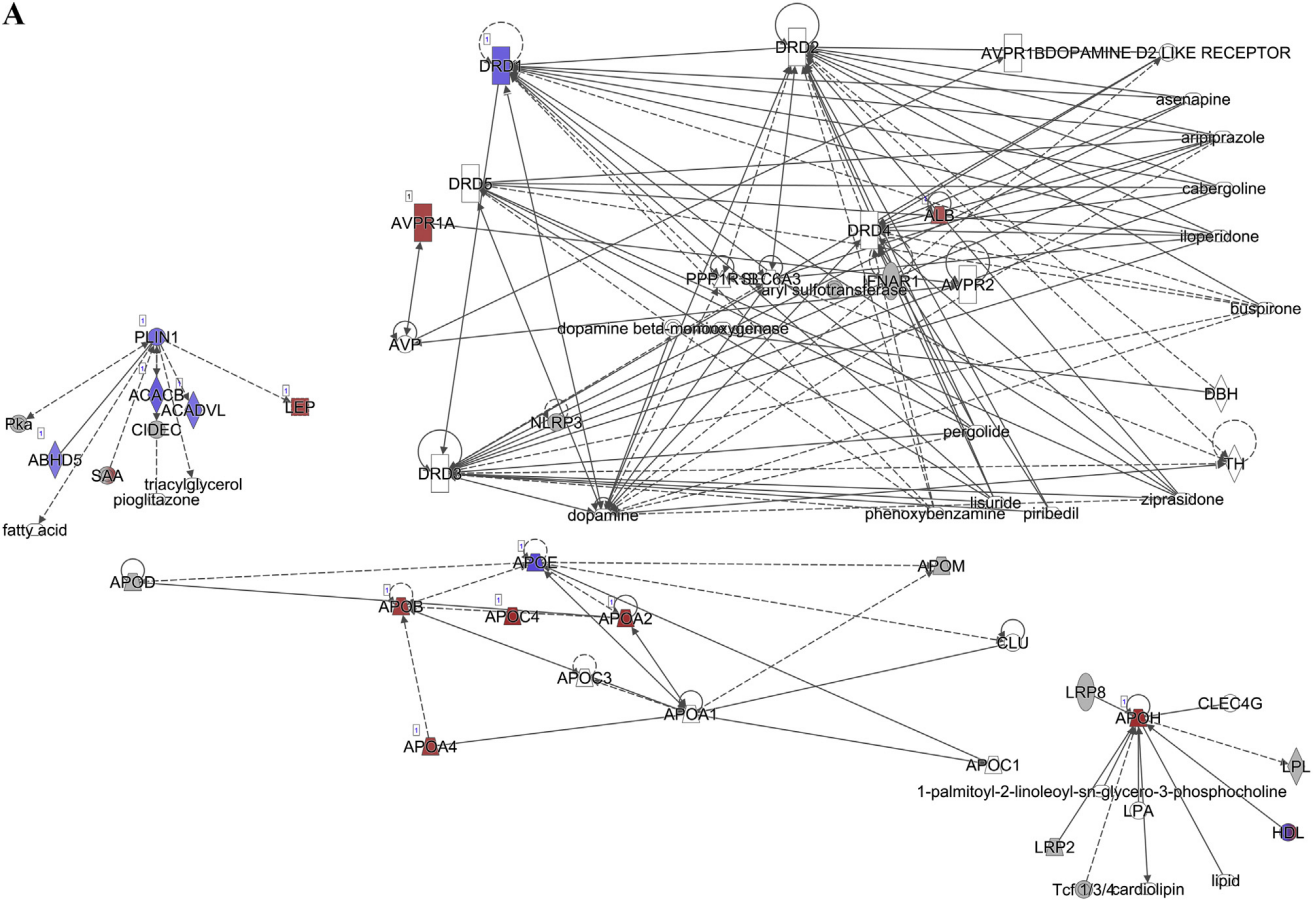

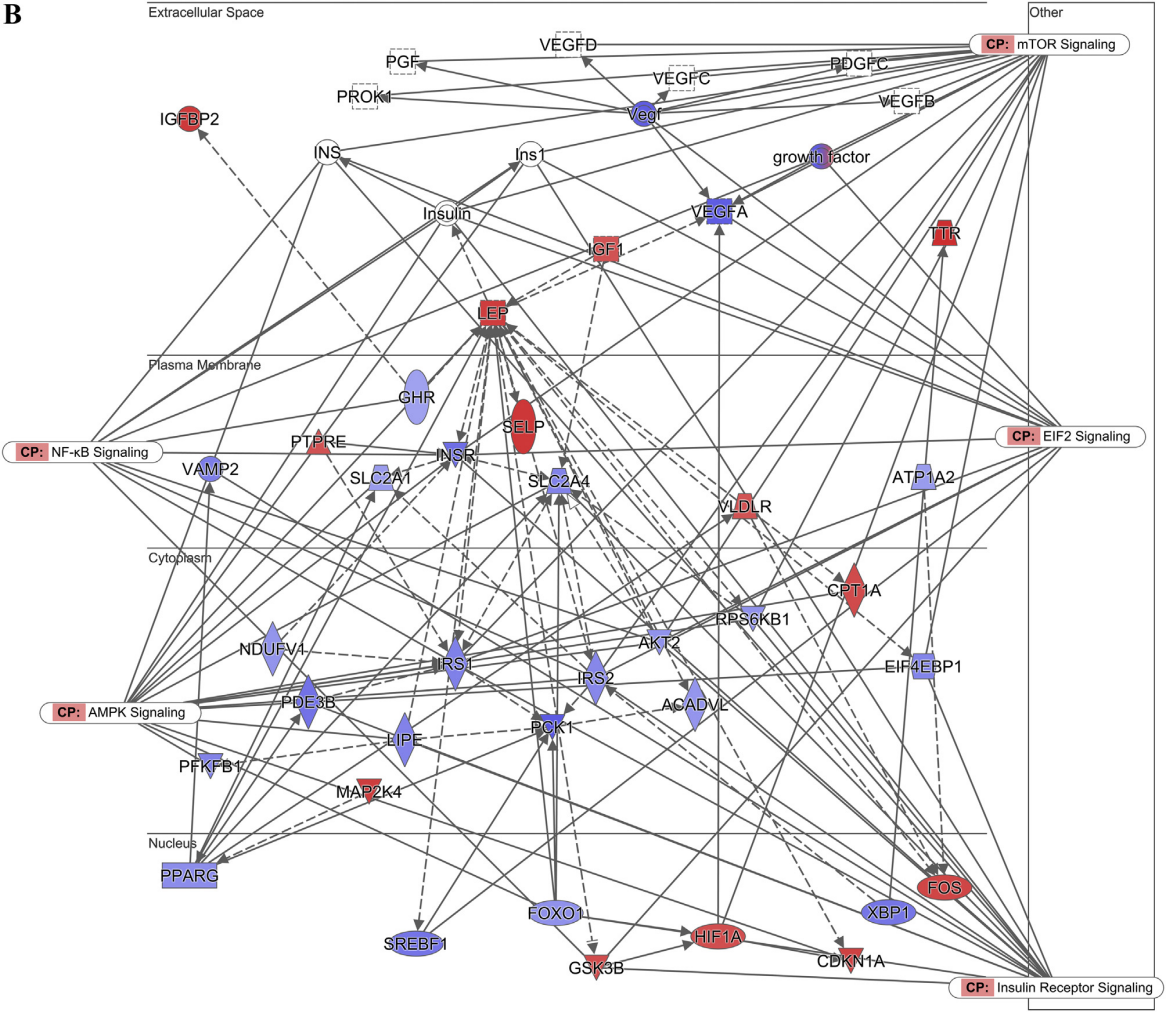

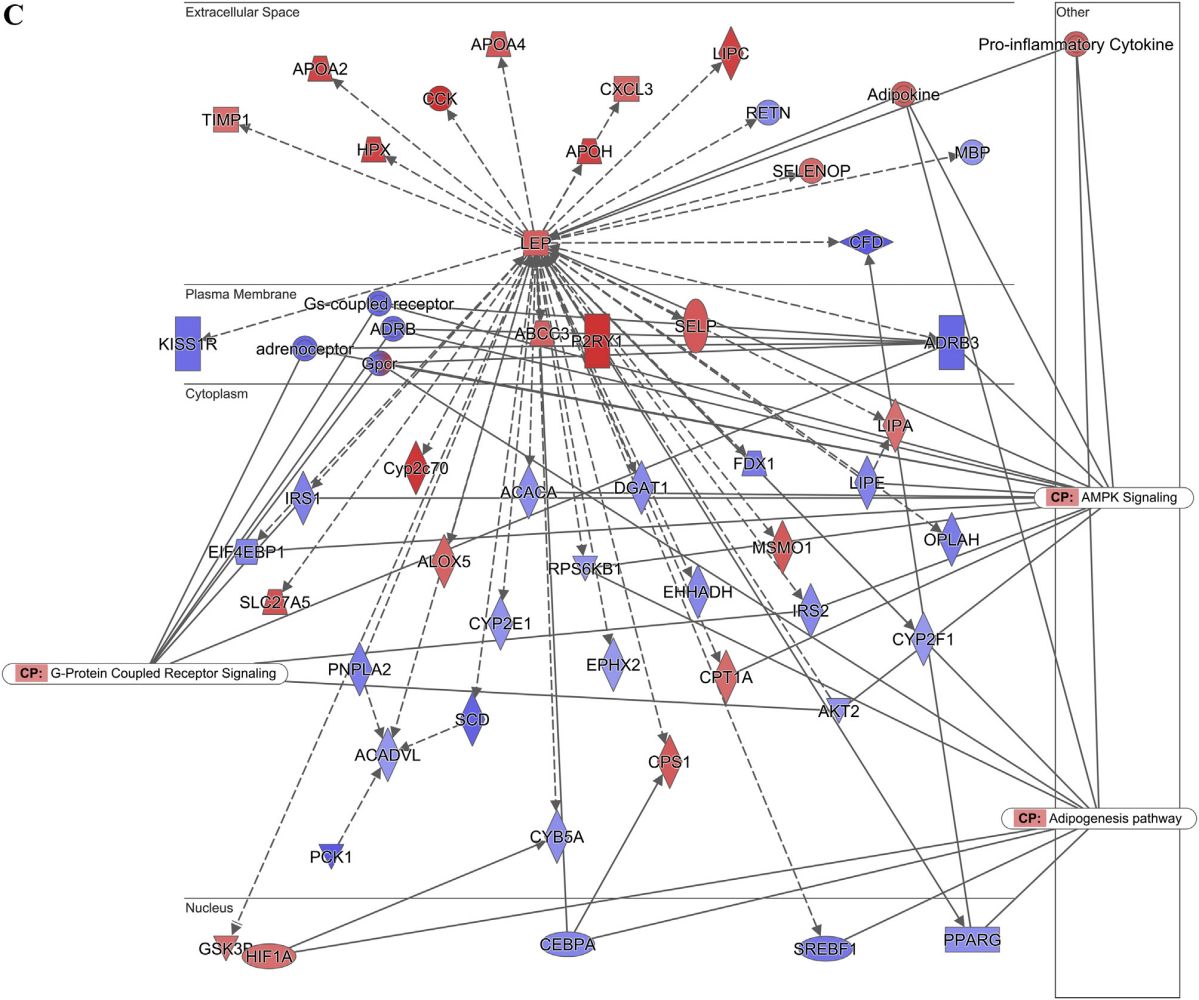

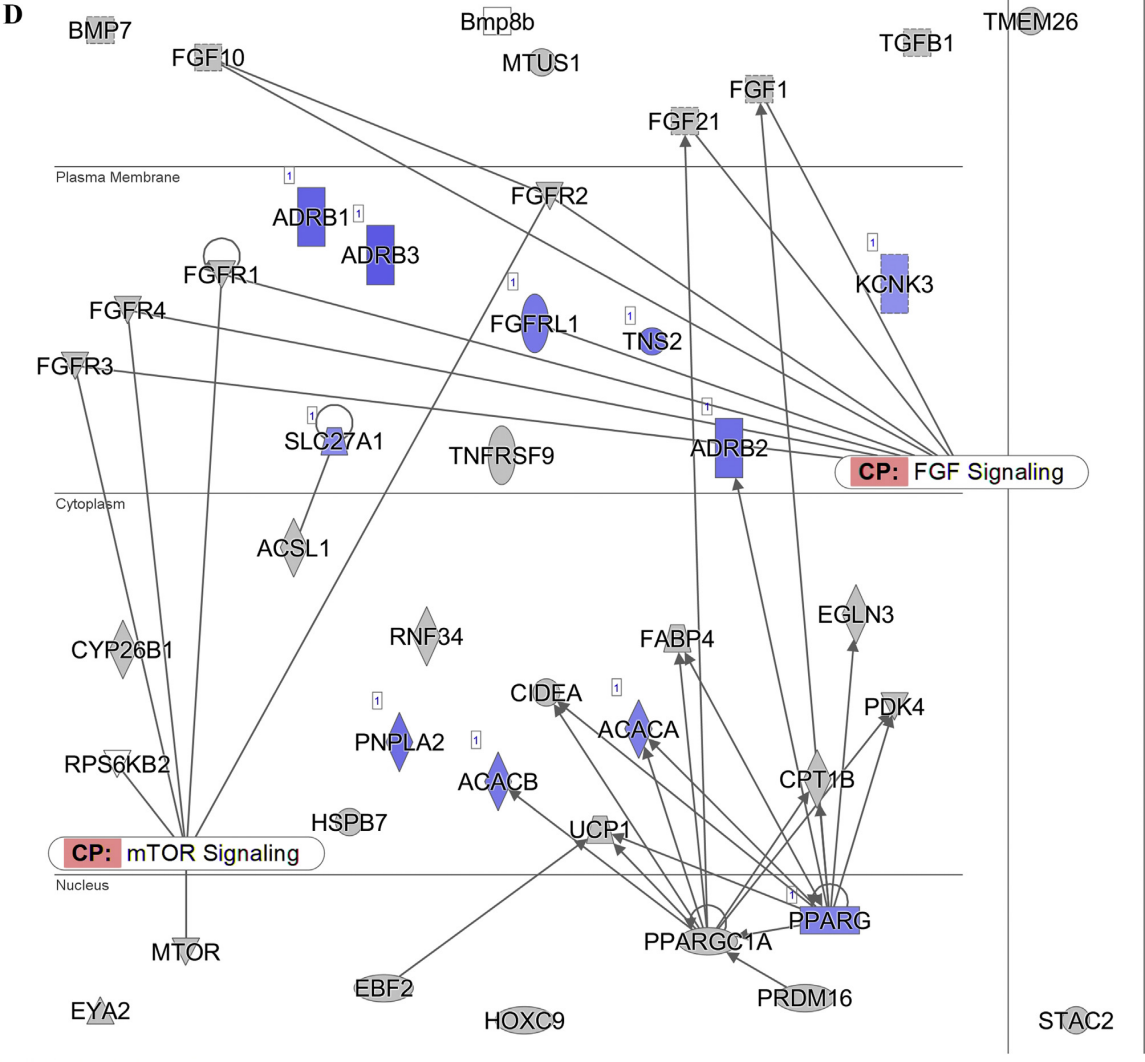
Diagram showing the correlation of the gene expression against the fasting insulin levels in the subcutaneous white adipose tissue of the C57BL/6 mice. (A) Lipolysis pathway-related genes. (B) Insulin signaling pathway-related genes. (C) Leptin signaling-pathway related genes. (D) Browning pathway-related genes. Red indicates the positive and blue indicates the negative correlations with the fasting insulin levels (p < 0.05). The color intensity is related to the absolute values of the correlation coefficients. Gray indicates no significance. A total of 24 pooled samples were used in the analysis across 24 diets, and each sample was pooled from 6 mice. See also Table S1.

The pathway analysis using the Ingenuity Pathway Analysis (IPA) demonstrated that the lipolysis pathway was significantly negatively correlated with the increasing insulin levels (p = 7.08 × 10^−7^) (Figure 2A), with 6 genes significantly downregulated and 8 genes significantly upregulated. The downregulated genes included *Apoe*, *Acacb*, *Drd1*, *Plin1*, *Acadvl*, *Cidec*, and *Abhd5*, and the upregulated genes were *Alb*, *Abpr1a*, *Lep*, *Apoa4*, *Apob*, *Apoa2*, *Apoh*, and *Apoc4* (for the full list, see Table S1). In contrast, the lipogenesis pathway did not appear on the list of significantly affected pathways.

The pathway analysis using the RNA-seq correlation data also demonstrated that the sWAT insulin signaling pathway was significantly inhibited in relation to the increasing insulin levels (p = 6.31 × 10^−8^) (Figure 2B), with 24 genes significantly downregulated and 4 genes upregulated. *Pparγ*, *Insr*, *Ins1*, *Ins2*, *Ghr*, and other related genes were downregulated, while *Ttr*, *Nov*, *Map2k4*, and *Aqp9* were upregulated (for the full list, see Table S1). A limitation of the current study is that we did not measure the insulin signaling components at the protein level. The leptin signaling pathway was also significantly inhibited (p = 7.59 × 10^−7^) (Figure 2C). There were 36 significantly downregulated genes in this pathway, and 29 genes were significantly upregulated. The full list of the altered genes is presented in Table S1.

**Figure 3:**
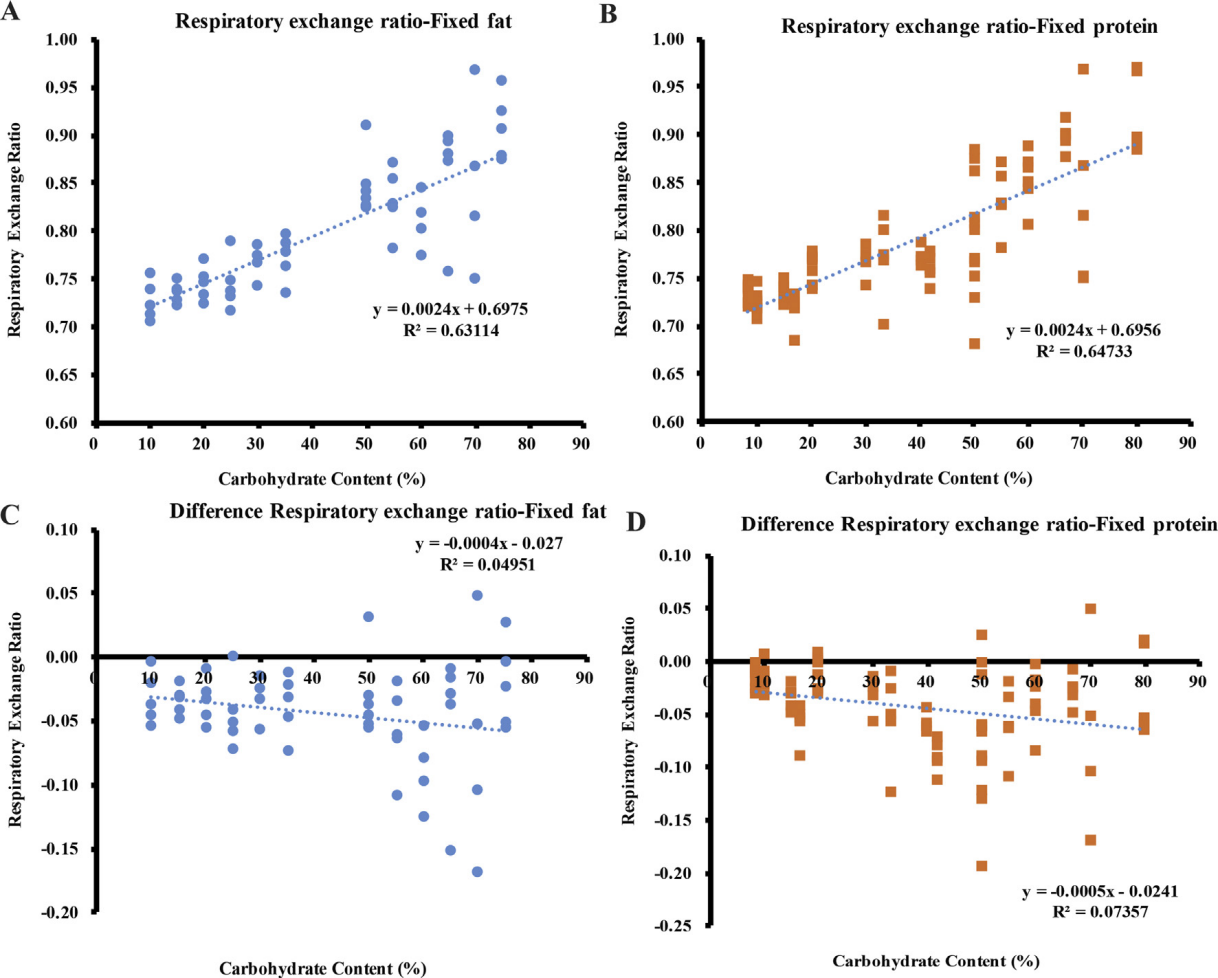
The respiratory exchange ratio (RER) of the mice following 10 weeks on experimental diets. The values were the measured values of each individual. The RER of the mice (A) when the dietary fat was fixed and (B) when the dietary protein was fixed. The discrepancy of the RER and FQ (C) when the dietary fat was fixed and (D) when the dietary protein was fixed. A total of 120 mice were used, with 4–7 mice per diet.

The RER increased significantly with the increasing dietary carbohydrate content but remained less than 1.0 regardless of the dietary fat (linear regression: F_1,57_ = 97.53, R^2^ = 0.631, p = 5.94 × 10^−14^) or protein content (linear regression: F_1,86_ = 157.86, R^2^ = 0.647, p < 1.0 × 10^−16^) (Figure 3A,B). The discrepancy between the RER and FQ was negatively related to the increases in the dietary carbohydrate content, either when the dietary fat was fixed (F_1, 57_ = 2.97, p = 0.090, R^2^ = 0.045) (Figure 3C) or when the dietary protein content was fixed (F_1, 86_ = 6.83, p = 0.011, R^2^ = 0.074) (Figure 3D).

A significantly positive non-linear relationship was observed between the body fat mass and fasting insulin levels independent of the dietary composition (polynomial regression: F_2,113_ = 59.49, R^2^ = 0.513, p < 0.001) (Figure S1A). Although the elevated fasting insulin was correlated with inhibited lipolysis signaling, insulin signaling, leptin signaling, and increased body fat mass that appeared to support the CIM's prediction, the elevated fasting insulin was related to the lower dietary carbohydrate content (see prediction A), and overall the model was not supported.

We then normalized the body fat mass in relation to the fasting insulin levels and performed a regression analysis between the residual body fat mass and dietary carbohydrate content. A significant negative relationship was observed between the residual body fat mass and dietary carbohydrate content (linear regression: F_1,114_ = 31.16, R^2^ = 0.215, p = 1.63 × 10^−7^) (Figure S1B). This demonstrated that the dietary carbohydrate content was negatively related to the body fat mass after normalization for the effect of the insulin levels. A multiple linear regression was then performed with the body fat mass as the dependent variable and both the fasting insulin and dietary carbohydrate content as the independent predictors (F_2,113_ = 65.76, R^2^ = 0.5297, p < 1.0 × 10^−16^). The body fat was significantly positively associated with the fasting insulin (R^2^ = 0.3804, p = 4.75 × 10^−10^) and negatively associated with the dietary carbohydrate (R^2^ = 0.1493, p = 1.53 × 10^−8^).

### 3.3 Prediction C): the increasing dietary carbohydrate content did not lead to greater food intake or lowered energy expenditure

**Figure 4:**
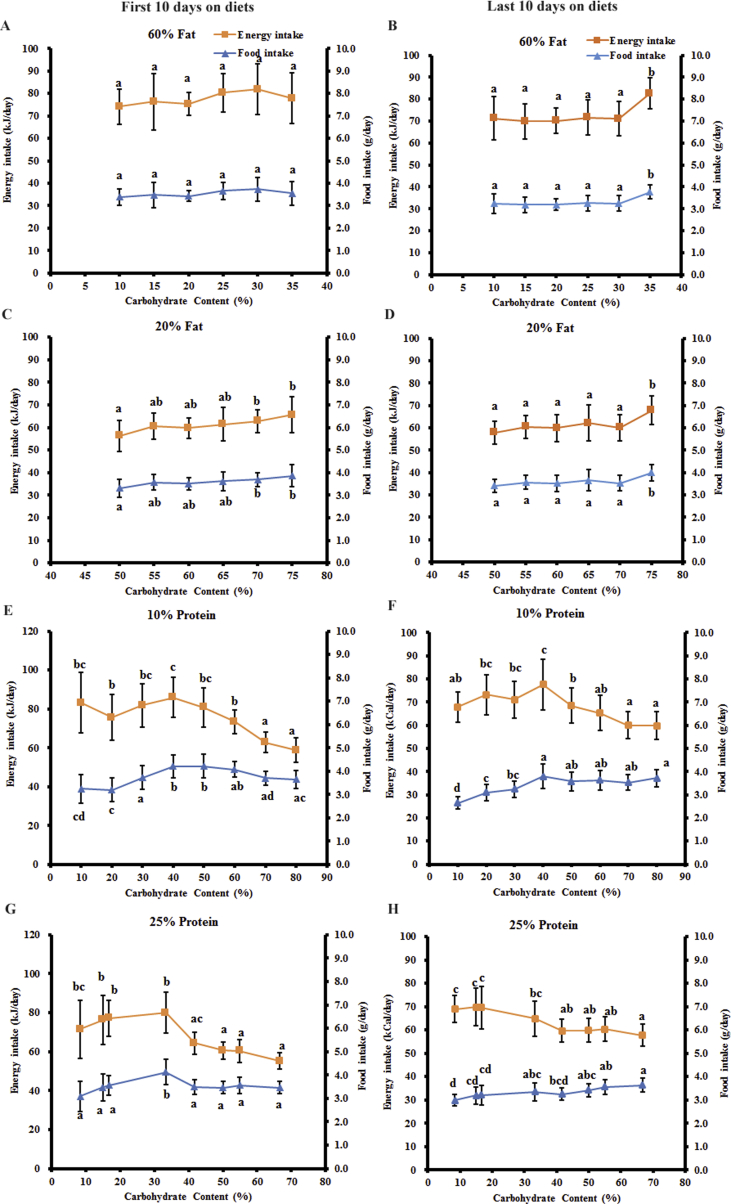
The energy/food intake of the mice following exposure to experimental diets. All of the values are presented as mean ± SD. (A) The energy/food intake of the mice fed diets with variable carbohydrate and fixed 60% fat. (B) The energy/food intake of the mice fed diets with variable carbohydrate and fixed 20% fat. (C) The energy/food intake of the mice fed diets with variable carbohydrate and fixed 10% protein. (D) The energy/food intake of the mice fed diets with variable carbohydrate and fixed 25% protein. a, b, c, and d represent p < 0.05. Groups with at least one same letter were not significantly different.

Food intake was measured daily over the entire 12-week dietary exposure period. The average food intake over the first and last 10 days were then used for comparison between the different diet groups. Similar trends were observed in the average food/energy intake over the first 10 days and last 10 days (Figure 4). Over the first 10 days following exposure, there was no difference in both the food intake and energy intake of the mice fed diets with variable carbohydrate content when the dietary fat content was fixed at 60% (ANOVA: p = 0.101 for food intake, p = 0.101 for energy intake) (Figure 4A). When the dietary fat content was fixed at 20%, there was no significant trend in both the food intake and energy intake when the carbohydrate level increased from 55% to 75%, except the significantly lower food/energy intake when the carbohydrate content was 50% than 70% and 75% carbohydrate (ANOVA: p = 5.16 × 10^−4^ for food intake, p = 5.07 × 10^−4^ for energy intake) (Figure 4C). When the dietary protein content was fixed at 10%, the energy intake of the mice decreased by 29.4% when the carbohydrate content increased from 10% to 80% (ANOVA: p = 2.61 × 10^−18^). The food intake increased by 30.2% when the dietary carbohydrate content increased from 10% to 50% and then decreased by 13.5% when the carbohydrate content increased to 80% (ANOVA: p = 5.23 × 10^−14^) (Figure 4E). When the dietary protein content was fixed at 25%, the energy intake of the mice decreased by 22.8% when the carbohydrate content increased from 8.4% to 66.7% (ANOVA: p = 2.10 × 10^−18^). There was no overall difference in the food intake when the carbohydrate content increased from 8.4% to 66.7%, except the significantly higher food intake when the carbohydrate content was 33.3% (ANOVA: p = 8.22 × 10^−8^) (Figure 4G).

Over the last 10 days following the 12-week exposure to the experimental diets, the trends in the food/energy intake of the mice on all 4 series of diets were similar to those of the first 10 days. When the dietary fat content was fixed at 60%, no difference was observed in the energy intake or food intake of the mice when the carbohydrate content increased from 10% to 30%, except the significantly higher energy intake/food intake when the carbohydrate content was 35% and the protein content was 5% (ANOVA: p = 1.40 × 10^−6^ for energy intake, p = 1.45 × 10^−6^ for food intake) (Figure 4B). Similarly, when the dietary fat content was fixed at 20%, there was also no difference in the energy intake or food intake of the mice when the carbohydrate content increased from 50% to 70%, except the significantly higher energy intake/food intake when the carbohydrate content was 75% and the protein content was 5% (ANOVA: p = 2.52 × 10^−5^ for energy intake, p = 2.57 × 10^−5^ for food intake) (Figure 4D). When the dietary protein content was fixed at 10%, the food intake of the mice increased when the carbohydrate content increased from 10% to 40% and then remained constant when the carbohydrate content increased to 80% (ANOVA: p = 8.71 × 10^−17^). The energy intake of the mice also increased when the carbohydrate content increased from 10% to 40%; however, it then decreased by 11.9% when the carbohydrate content increased to 80% (ANOVA: p = 3.24 × 10^−12^) (Figure 4F). When the dietary protein content was fixed at 25%, the food intake of the mice increased significantly when the carbohydrate content increased from 8.4% to 66.7% (ANOVA: p = 5.36 × 10^−8^) (Figure 4H). However, the energy intake decreased by 16.2% when the carbohydrate content increased from 8.4% to 66.7% (ANOVA: p = 6.26 × 10^−12^) (Figure 4H). As there was no increase in the energy intake with the increasing carbohydrate content when the dietary fat content was fixed, and the energy intake was higher when the dietary fat was fixed at 60% than 20%, the decrease in the energy intake when the dietary protein was fixed may be related to the change in the dietary fat content rather than the carbohydrate content.

**Figure 5:**
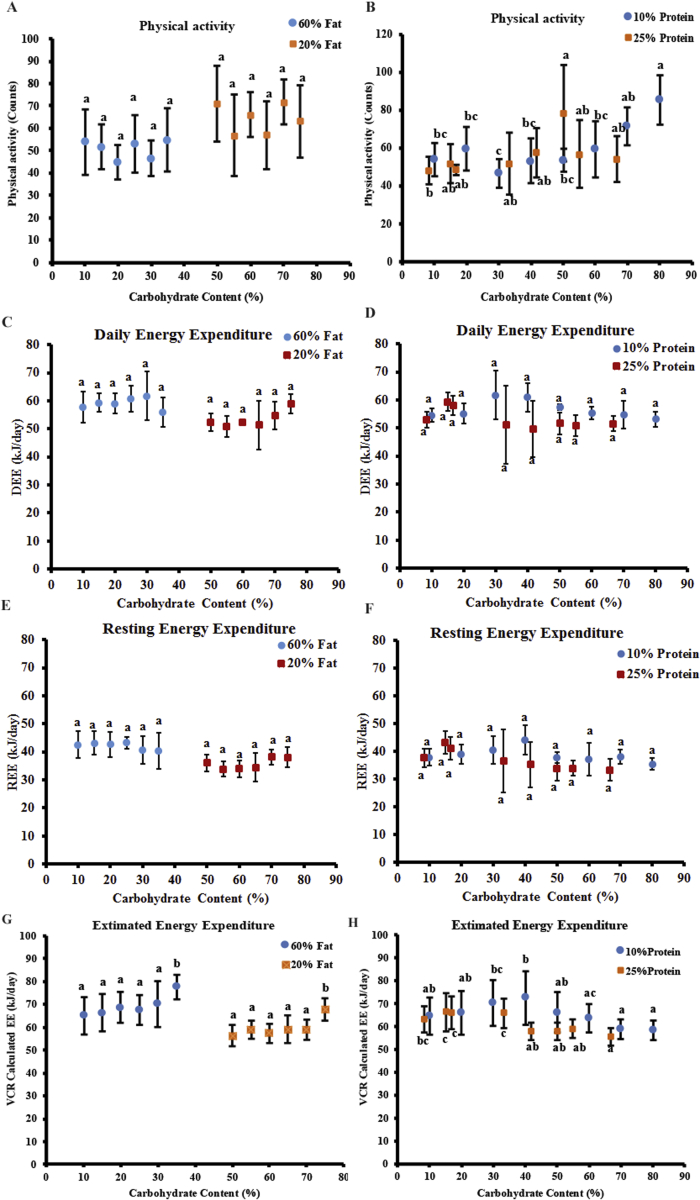
The energy expenditure of the mice following exposure to experimental diets. All of the values are presented as mean ± SD. The daily energy expenditure (DEE) of the mice fed diets with (A) fixed 60% or 20% fat and (B) fixed 10% or 25% protein. The REE of the mice fed diets with (C) fixed 60% or 20% fat and (D) fixed 10% or 25% protein. The physical activity of the mice fed diets with (E) fixed 60% or 20% fat and (F) fixed 10% or 25% protein. A total of 120 mice were used, with 4–7 mice per diet. The estimated energy expenditure of the mice fed diets with (G) fixed 60% or 20% fat and (H) fixed 10% or 25% protein throughout the experimental period. A total of 480 mice were used, with 20 mice per diet. a, b, c, and d represent p < 0.05. Groups with at least one same letter were not significantly different.

We measured the physical activity and energy expenditure of the mice using a TSE PhenoMaster system after a 10-week exposure to the experimental diets. There was no significant trend in the physical activity across the mice fed diets with different carbohydrate content when the fat content was fixed (ANOVA: p = 0.713 for 60% fat, p = 0.452 for 20% fat) (Figure 5A). When the protein content was fixed at 10%, no significant differences were observed in the physical activity of the mice fed diets with different carbohydrate content, except higher physical activity of the mice on diets with 70%/80% carbohydrate than with 30% carbohydrate (ANOVA: p = 2.71 × 10^−5^) (Figure 5B). When the protein content was fixed at 25%, there was also no significant differences in the physical activity of the mice on diets with different carbohydrate content, except between diets with 50% carbohydrate and 8.4% carbohydrate (ANOVA: p = 0.031) (Figure 5B).

Both the daily energy expenditure (DEE) (ANOVA: p = 0.603 for 60% fat, p = 0.128 for 20% fat, p = 0.017 for 10% protein, p = 0.24 for 25% protein) and resting energy expenditure (REE) (ANOVA: p = 0.874 for 60% fat, p = 0.214 for 20% fat, p = 0.060 for 10% protein, p = 0.093 for 25% protein) of the mice showed no changes in relation to the dietary carbohydrate content independent of the dietary fat and protein content (Figure 5C–F). We also estimated the average energy expenditure of the mice throughout the experimental period using an energy balance software algorithm [31,32]. When the dietary fat content was fixed at 60% or 20%, there was no difference in the average energy expenditure of the mice fed diets with variable carbohydrate content, only significantly higher energy expenditure when the carbohydrate content was 35% or 75% (ANOVA: p = 7.57 × 10^−6^ for 60% fat, p = 2.74 × 10^−11^ for 20% fat) (Figure 5G). When the protein content was fixed at 10% or 25%, the average energy expenditure remained constant when the dietary carbohydrate content increased from 10% to 40%, which then decreased when the carbohydrate content increased to 80% (ANOVA: p = 1.86 × 10^−7^ for 10% protein, p = 4.28 × 10^−12^ for 25% protein) (Figure 5H).

### 3.4 Prediction D): elevated fasting insulin was correlated with the inhibition of pathways regulating food intake in the brain. Adipose tissue browning was inhibited, although the overall daily energy expenditure was not changed

**Figure 6:**
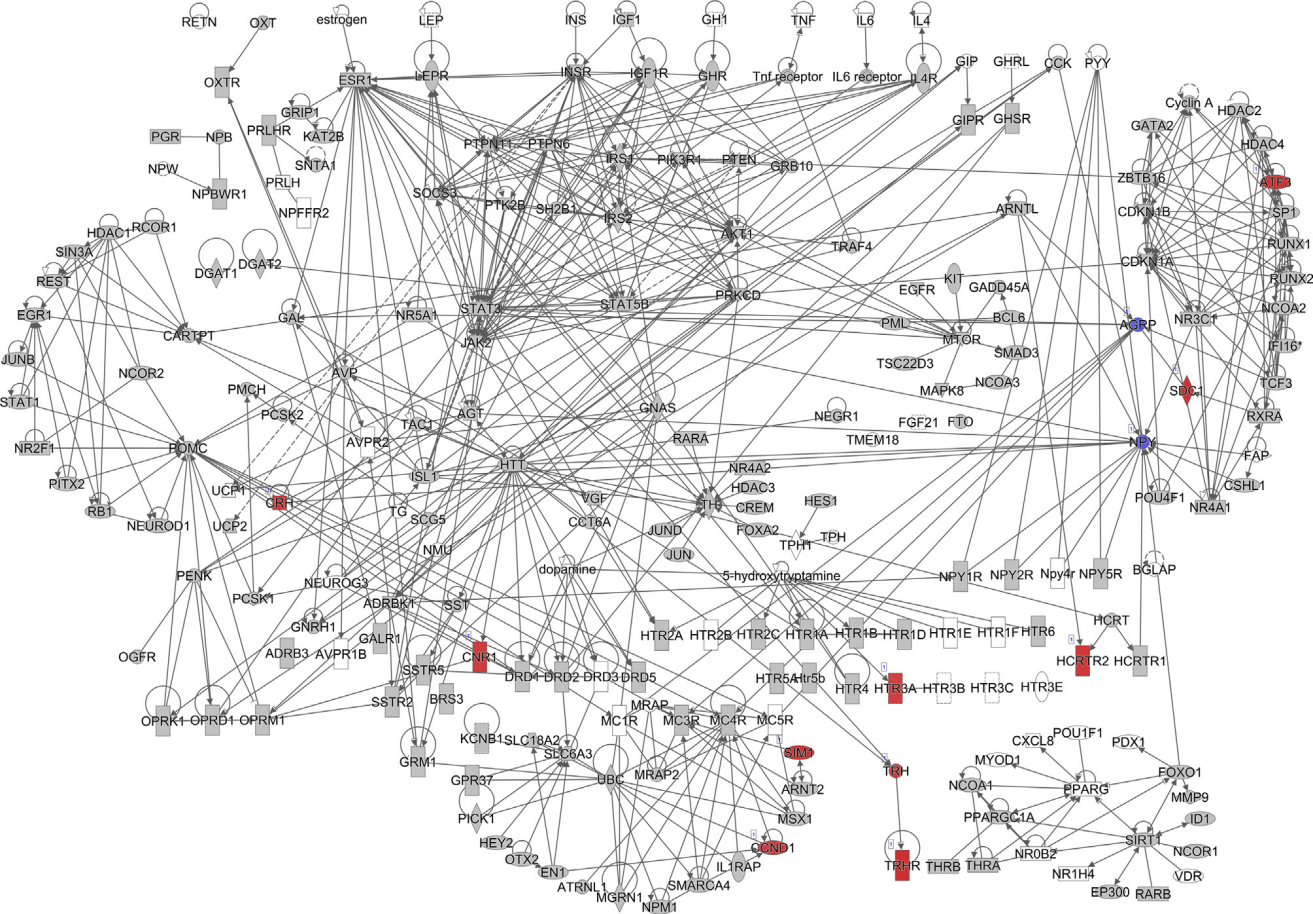
Hunger pathway diagram showing the correlation of the gene expression against the fasting insulin levels in the hypothalamus of the C57BL/6 mice. Red indicates the positive and blue indicates the negative correlations with the fasting insulin levels (p < 0.05). The color intensity is related to the absolute values of the correlation coefficients. Gray indicates no significance. A total of 48 pooled samples were used in the analysis across 24 diets, and each sample was pooled from 4 mice. See also Table S2.

We also calculated Pearson's correlation between the fasting insulin levels and gene expression profiles of the hypothalamus from RNA sequencing (RNA-seq) of the mice fed experimental diets for 12 weeks to investigate the effect of the fasting insulin levels on the canonical hunger signaling pathways. There were 347 genes in the hypothalamus significantly correlated with the fasting insulin levels in the mice fed diets with variable carbohydrate content (Table S2), of which 316 genes were positively correlated and the other 31 were negatively correlated with the fasting insulin levels. The pathway analysis showed that 2/228 genes were significantly downregulated and 10/228 genes were significantly upregulated in the hunger signaling pathway (p = 1.66 × 10^−4^) (Figure 6A). *Agrp* and *Npy* were both significantly downregulated, while *Htr3a*, *Cnr1*, *Trh*, *Ccnd1*, *Crh*, *Hcrtr2*, *Sdc1*, *Aft3*, *Trhr*, and *Sim1* were significantly upregulated (for the full list, see Table S2). *Agrp* and *Npy* are neuropeptides that potently stimulate food intake. The pattern of changes in these genes with the circulating insulin levels implies that higher peripheral insulin levels inhibit rather than stimulate intake.

In the adipose browning pathway, 11/42 genes were significantly negatively correlated with the fasting insulin levels (p = 1.66 × 10^−2^) (for the full list, see Table S1). These significantly downregulated genes included *Adrb3*, *Adrb1*, *Pnpla2*, *Adrb2*, *Tns2*, *Acacb*, *Fgfrl1*, *Acaca*, *Pparγ*, *Slc27a1*, and *Kcnk3* (Figure 2D).

### 3.5 Prediction E): there was no positive energy balance, and thus there was no increase in the body adiposity and overall body mass with the increasing dietary carbohydrate content

**Figure 7:**
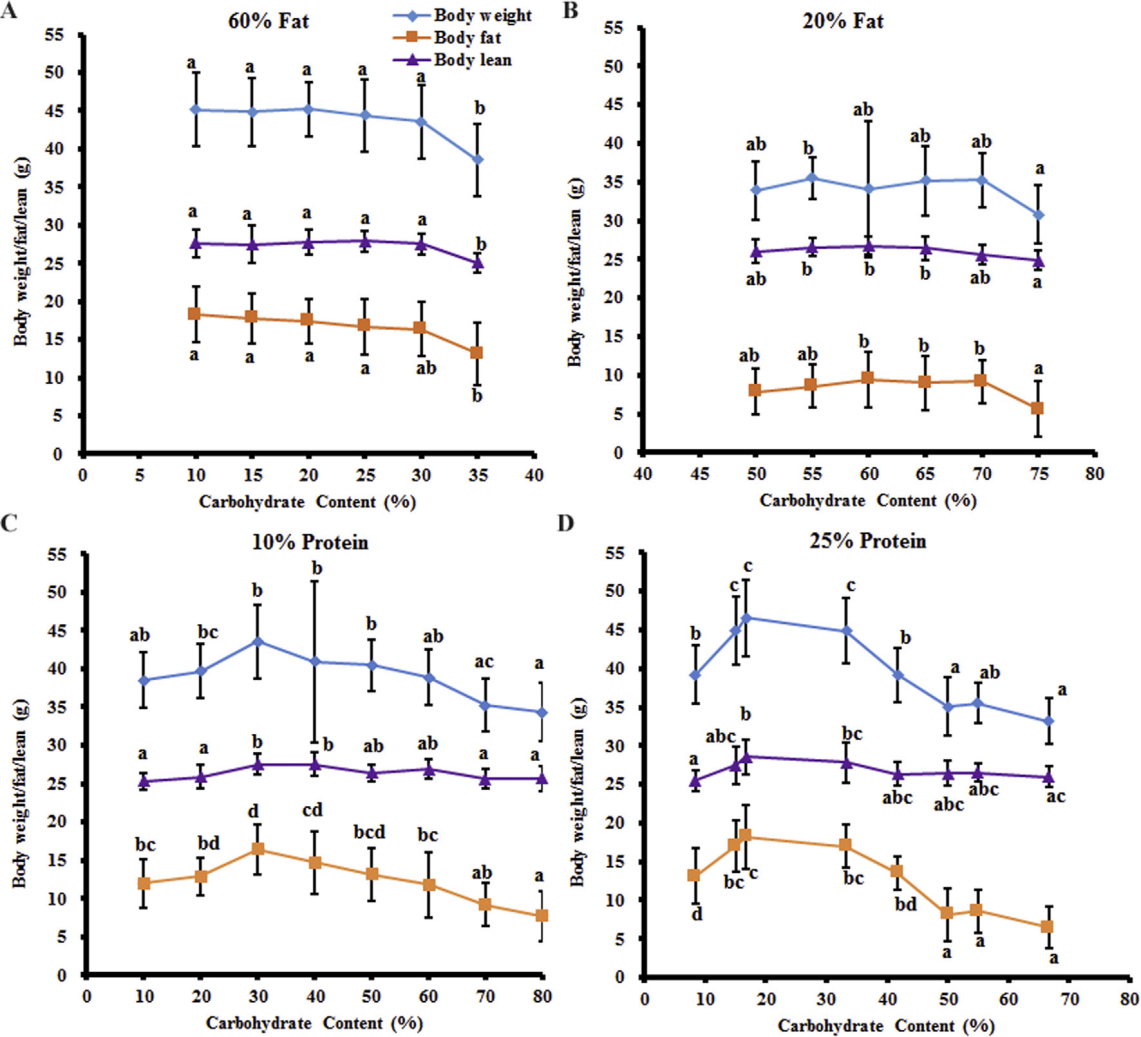
The body weight, fat, and lean mass of the mice fed experimental diets. All of the values are presented as mean ± SD. (A) Fixed 60% fat. (B) Fixed 20% fat. (C) Fixed 10% protein. (D) Fixed 25% protein. A total of 480 mice were used, with 20 mice per diet. a, b, c, and d represent p < 0.05. Groups with at least one same letter were not significant.

When the dietary fat content was maintained constant at 60% or 20%, the body mass (ANOVA: p = 1.92 × 10^−5^ for 60% fat, p = 0.024 for 20% fat), fat mass (ANOVA: p = 2.30 × 10^−4^ for 60% fat, p = 0.005 for 20% fat), and lean mass (ANOVA: p = 2.14 × 10^−5^ for 60% fat, p = 0.0015 for 20% fat) did not change with the increasing dietary carbohydrate content from 10% to 30% (60% fat) or 50%–70% (20% fat), with only significantly lower masses with 35% or 75% carbohydrate content (Figure 7A,B). In the fixed 10% protein diet group, the body mass increased by 13.2%, fat mass by 37.0%, and lean mass by 8.6% when the dietary carbohydrate content increased from 10% to 30% and then decreased by 21.2%, 52.9%, and 6.8%, respectively, when the dietary carbohydrate content increased from 30% to 80% (ANOVA: p = 6.29 × 10^−6^ for body mass; ANOVA: p = 3.48 × 10^−10^ for fat mass, p = 8.01 × 10^−6^ for lean mass) (Figure 7C). Similarly, when the protein content was fixed at 25%, the body mass increased significantly by 18.7%, fat mass by 38.9%, and lean mass by 12.0% when the dietary carbohydrate content increased from 8.4% to 20% and then decreased by 28.7%, 64.5%, and 9.1%, respectively, when the carbohydrate content increased to 66.6% (ANOVA: p = 4.56 × 10^−24^ for body mass, p = 1.59 × 10^−22^ for fat mass, p = 2.70 × 10^−4^ for lean mass). The changes in the body mass of the mice were mainly from the increases in the fat mass despite the slight difference in the lean mass when fed diets with variable carbohydrate content independent of the protein content (Figure 7D).

In summary, we tested five predictions of the carbohydrate-insulin model in this study. The predictions that the post-prandial insulin levels would increase and the fasting glucose would decrease in relation to the dietary carbohydrate content were supported. In relation to the elevated fasting insulin, the lipolysis and leptin signaling pathways in the sWAT were inhibited, supporting the CIM's predicted impact of high insulin. However, the higher fasting insulin was not related to the higher carbohydrate levels; therefore, the overall fasting insulin and dietary carbohydrate patterns did not support the CIM. Moreover, the hypothalamic hunger pathway was inhibited in relation to the elevated fasting insulin. The energy intake did not increase in relation to the increased dietary carbohydrate. The browning pathway in the sWAT was inhibited at higher insulin levels, but the daily energy expenditure did not change. There was no positive energy balance when the dietary carbohydrate content increased, and hence no overall increase in the adiposity and body weight was observed. Overall, two of the predictions were partially supported (and hence also partially not supported), while three of the predictions were not supported. We therefore conclude that the model was not supported by these data in mice.

## 4 Discussion

### 4.1 Prediction A): the post-prandial insulin was positively associated with the dietary carbohydrate intake, supporting the CIM, but the fasting insulin was negatively associated with the dietary carbohydrate content

The post-prandial insulin levels of the mice increased with the increasing dietary carbohydrate intake, consistent with a previous study of mice comparing just two diets with 6% and 70% carbohydrate [17]. Increased dietary carbohydrate also led to increased daily insulin secretion in humans [19]. In both mice and humans, blood glucose levels rise in response to bolus doses of glucose as observed in the classical glucose tolerance test. It may be surprising therefore to note that the post-prandial glucose levels observed herein were unrelated to the carbohydrate intake. However, in the current study, the carbohydrate was ingested at the same time as other macronutrients, which may have affected the blood glucose response [[38], [39], [40], [41], [42], [43]]. Moreover, recent research has demonstrated that blood glucose is elevated by protein intake in humans [44,45].

The decrease in the fasting insulin levels with the elevated dietary carbohydrate content was also consistent with a previous study which found that fasting insulin levels in mice decreased with the increasing dietary carbohydrate content after 8 weeks on experimental diets [46]. In contrast, higher fasting insulin levels were observed in mice on a diet with 6% carbohydrate compared with a chow diet that had 70% carbohydrate after 6–7 weeks of exposure [17]. Similarly, the fasting insulin levels of rats were not strongly linked to dietary carbohydrate content between 5% and 60% carbohydrate [18]. Another laboratory also reported significantly lower fasting insulin levels in rats fed a diet with 1.3% carbohydrate than those fed 58% carbohydrate [15,16]. A recent study demonstrated that the fasting insulin levels of mice fed a diet with 46% sucrose (62.6% total carbohydrate) were not different from those on a diet with 7.6% sucrose (62.3% total carbohydrate) after 20 weeks of exposure [47], also indicating no strong impact of the carbohydrate composition on the fasting insulin levels. The fasting glucose levels decreased in relation to the elevated dietary carbohydrate content in our study. This was consistent with a previous report that the blood glucose of overnight fasted mice on a 6% carbohydrate diet was significantly higher than that of 70% carbohydrate [17]. In contrast, Caton et al. reported significantly lower blood glucose levels in mice fed a diet with 1.3% carbohydrate than with 58% carbohydrate following a 6 h fast [15]. Several other studies in rats [18] and mice [46] found no effects of the dietary carbohydrate content on the fasting glucose levels.

Overall, our data, while not completely consistent with previous studies of rodents, supported the CIM's prediction that post-prandial insulin would rise after higher carbohydrate consumption and this would subsequently lead to a “starvation state” reflected by lower circulating fasting glucose levels in relation to the dietary carbohydrate content. However, while these two results supported the CIM's prediction, the fasting insulin data refuted the prediction.

### 4.2 Prediction B): the elevated fasting insulin level was related to the inhibited lipolysis and leptin signaling pathways, yet it contradicted the CIM because of the negative relationship between the fasting insulin and dietary carbohydrate content

In relation to the increase in fasting insulin levels, the lipolysis and leptin signaling pathways in the sWAT of the mice were inhibited. Previous studies indicated that the inhibition of both pathways was associated with fat storage [48], indicating a positive relationship between the fasting insulin levels and body fat mass. Consistent with this, we also observed a positive relationship between the body fat mass and fasting insulin levels (Figure S1A). However, we did not observe changes in the lipogenesis pathway in the sWAT that were predicted by the CIM. This was consistent with the measured respiratory exchange ratio (RER). High levels of conversion of glucose into lipids can lead to an RER >1.0, indicating net lipogenesis [49,50]. As the dietary carbohydrate content increased, the RER values of the mice gradually increased to approach 1.0 in this study, indicating normal macronutrient metabolism and no lipogenesis stimulation. The RER was lower than the FQ in the mice fed all of the diets with variable carbohydrate content, and the absolute discrepancy increased with the increased carbohydrate content. This may indicate the mobilization of energy from body fat [51] rather than the storage of body fat as predicted by the CIM. In this study, the variability in the fasting insulin was greater when the carbohydrate content was lower, while the variability in the RER was smaller. When the carbohydrate content was lower, the body weight and body fat of the mice were also more variable (Figure 7, Figure S1), which may explain the greater variability in the fasting insulin levels. V_O2_ and the RER were more variable during the dark and light phases and the RER was more variable between the dark and light phases when the female mice were fed a diet with 70% carbohydrate than a diet with 35% carbohydrate [52]. In humans, the RER increased during exercise compared to the rest state, demonstrating a link between the RER and physical activity [53]. In our study, the variability in the physical activity tended to be higher in the high carbohydrate conditions, independent of the dietary protein and fat content. The high dietary carbohydrate content and greater variability in physical activity may have led to the higher variability in the RER under high carbohydrate conditions.

The effect of insulin levels on body weight is controversial and has long been debated [54,55]. It is widely accepted that increasing body adiposity is related to elevated insulin resistance, and in turn, the insulin resistance compensatory response increases insulin levels and decreases insulin sensitivity under both basal and stimulated conditions [[56], [57], [58], [59]]. In the obese state, adipose tissues release more non-esterified fatty acids, glycerol, hormones, pro-inflammatory cytokines, and many other factors that are involved in the development of insulin resistance [60,61]. Insulin resistance triggers hyperinsulinemia, and hyperinsulinemia in turn causes insulin resistance [61,62]. However, it was recently suggested that hyperinsulinemia may be the primary disruption in obesity, which then drives insulin resistance [61,[63], [64], [65], [66]]. Studies used mice lacking *Ins1* and/or *Ins2* genes to reduce circulating insulin, and the reduction in insulin led to attenuated body weight and adiposity gain in the mice [[63], [64], [65], [66]]. Moreover, in obese mice, reducing the circulating insulin levels via genetic manipulation led to significant body weight loss within 5 weeks and reversed the existing obesity [67]. These studies used mice null for the *Ins1* or *Ins2* genes, reduced insulin levels by partial null *Ins2* or *Ins1* genes, and showed that lower insulin was associated with decreased adiposity. This is not the same as showing that elevated insulin resulted in elevated adiposity. Studies of gain of function mutations or insulin infusion also did not result in elevated body fat [[68], [69], [70], [71]], but this could be because developing hypoglycemia produces confounding effects on the metabolic rate and food intake. The directionality of the causality between circulating insulin and adiposity therefore remains unsettled. These genetic studies confirm that lower insulin levels are linked to lower development of fat mass, consistent with the positive association between adiposity and fasting insulin levels observed herein. This would provide support for the CIM if the fasted insulin levels increased in relation to the dietary carbohydrate exposure, but as we (and other laboratories) have shown, the relation of fasting insulin to dietary carbohydrate content was the opposite over the range from 15% to 80% carbohydrate. In our study, there was also an inhibition of the insulin signaling pathway in relation to the elevated fasting insulin, which suggests a negative feedback of insulin on insulin signaling in subcutaneous white adipose tissue. It was previously reported that insulin signaling is required for lipid storage in fat, partly by inhibiting lipolysis [72], possibly indicating a mechanism against further body fat gain. Overall, this may indicate that increased insulin can lead to body fat gain via the inhibition of lipolysis and leptin signaling; however, a sustained increase in insulin also inhibits the insulin signaling pathway to prevent further body fat gain. This is consistent with previous reports of the beneficial and deleterious effects of insulin in obesity and other insulin-resistant syndromes [61].

The inhibition of the lipolysis and leptin signaling pathways by elevated fasting insulin supported the predicted effect of insulin, yet because of the negative relationship between dietary carbohydrate and insulin, the overall effect refuted the CIM's prediction. The inhibition of the insulin signaling pathway, in relation to the elevated fasting insulin, did not support the CIM's predicted insulin effect.

### 4.3 Prediction C): dietary carbohydrate was not a driver of energy intake or a factor affecting energy expenditure, in contrast with the CIM's predictions

In our study, the energy intake of the mice did not increase in relation to the elevation of the dietary carbohydrate content. As previously reported via the prevailing energy homeostatic model, energy intake is mainly regulated by energy demand [4], but this balance can be overridden by high dietary fat content due to hedonically driven eating in mice [27]. Even high dietary sucrose content (up to 73%) did not impact the energy intake in mice [73], although adding sucrose to their drinking water did have an impact [66,73]. These studies suggest that dietary carbohydrate is not a key driver of energy intake, and therefore is not the main factor that influences body adiposity.

The energy expenditure did not decrease in relation to the increased dietary carbohydrate content in this study, consistent with our prior report which demonstrated that increased sucrose content had no effect on energy expenditure [27,73]. This contradicts a previous study that demonstrated in mice with both normal weight and diet-induced obesity, a ketogenic diet with 0.76% carbohydrate led to higher energy expenditure [74]. Variable results have been reported on the impact of low carbohydrate diets on energy expenditure in humans. In overweight or obese adults, following 10%–15% weight loss, isocaloric diets with variable carbohydrate content resulted in decreases in both the resting energy expenditure and total energy expenditure (measured using doubly labeled water), although the decreases were greater with higher dietary carbohydrate [75]. In contrast, in a more recent study of overweight or obese men, the total energy expenditure (measured both in a metabolic chamber and using doubly labeled water) and sleeping energy expenditure was significantly increased, although the changes were marginal, when their diets were switched to a ketogenic diet [22]. These studies compared two or three diets, while we manipulated diets by fixing the protein or fat and included 24 diets in our research. In our study, there was no stimulation of intake and no reduction in energy expenditure and hence no positive energy balance when the dietary carbohydrate content increased, all contradicting the CIM's predictions.

### 4.4 Prediction D): higher fasting insulin was correlated with the inhibition of the hunger pathway rather than the CIM's predicted stimulation of the pathways. The white adipose tissue browning pathway was inhibited in relation to the increased insulin

We demonstrated that the higher fasting peripheral insulin levels were correlated with lower levels of *Npy* and *Agrp* expression in the hypothalamus, consistent with many previous studies [[76], [77], [78], [79], [80], [81]]. We also found that *Htr3a*, *Trh*, *Trhr*, and *Crh* were upregulated in relation to the fasting insulin levels. Central serotonin (5-HT) plays an anorexigenic role in the brain [82]. Together with the reductions in *Npy* and *Agrp*, the upregulation of *Htr3a*, one of the 5-HT receptors, may also be related to the observed reduction in the food intake. A previous study reported that the *Htr3a* gene was associated with the restricted type of anorexia nervosa, which is characterized by strict dieting and physical activity in humans [83]. *Trh* has an anorexigenic effect on the regulation of feeding behavior [84,85]. The *Trhr* gene has also been suggested to regulate some biological functions linked to energy balance, including an increase in thermogenesis, total energy expenditure, and lipid and carbohydrate catabolism [86,87]. *Crh* can reduce food intake and increase metabolic activity [[88], [89], [90], [91]]. A deficit of *Crh* activates *Agrp* neurons, which then decrease heat production in the BAT and browning of the WAT [91]. Therefore, the downregulation of *Npy* and *Agrp* and upregulation of *Htr3a*, *Trh*, *Trhr*, and *Crh* would all be expected to lead to a reduction in energy intake as the fasting insulin increased with the decreasing dietary carbohydrate. The changes in the genes contradicted the CIM's prediction that the elevated insulin in relation to the increased dietary carbohydrate should stimulate the pathways regulating food intake and therefore increase the energy intake in relation to the elevated dietary carbohydrate. In our study, there was no increase in the energy intake of the mice fed diets with increased carbohydrate under either fixed fat or protein content.

The adipose tissue browning pathway was inhibited as the fasting insulin levels increased, which was consistent with the results of a previous study in which enhanced browning was correlated with genetically reduced insulin levels [63]. However, this change in the browning level would only be consistent with the CIM's predictions if the fasting insulin was positively associated with the dietary carbohydrate content, which it was not. Moreover, the inhibition of the browning pathway was insufficient to alter the daily energy expenditure, consistent with previous research suggesting that this process is a relatively small contributor to overall energy demands [92]. As no changes were observed in the physical activity and resting energy expenditure of the mice in this study, if there was an impact of the inhibition of the browning pathway on the energy expenditure, it may have been compensated by elevated diet-induced thermogenesis.

### 4.5 Prediction E): contrary to the CIM's predictions, the increase in the dietary carbohydrate content did not lead to greater body adiposity or overall body weight, independent of the dietary fat and protein content

Previous diet intervention studies reported inconsistent results on body weight gain or loss in relation to variable dietary carbohydrate content. In obese New Zealand mice, a low carbohydrate diet (6% energy) increased weight gain and adipose tissue mass, although the fasting insulin levels of the mice were higher than mice on a chow diet with 70% carbohydrate [17]. In other studies, rats fed a low carbohydrate diet (1.3% energy) lost weight yet had increased fat mass, even in combination with forced daily exercise [15,16]. In diet-induced obese rats, no difference was observed in the body weight and energy intake of the rats between those fed a very low carbohydrate diet (5% energy) and a high carbohydrate diet (60% energy) over an 8-week period [18]. Most studies compared only two or three diets, either low carbohydrate or low fat with different compositions. In our study, we designed 24 different diets by manipulating their carbohydrate content from 8.4% to 80%, either at a fixed fat content or a fixed protein content, and also controlled the diets' fatty acid, carbohydrate, and protein compositions. Independent of whether the fat or protein levels were fixed, the body mass and fat mass of the mice did not increase when the dietary carbohydrate content increased. Therefore, contrary to the CIM's predictions, elevations in the dietary carbohydrate content did not lead to increased body mass and fat mass in the mice.

## 5 Conclusion

Overall, only the changes in the post-prandial insulin and fasting glucose followed the CIM's predictions in relation to the dietary carbohydrate. The fasting insulin, energy intake, energy expenditure, and body fat mass did not follow the trends predicted by the CIM. In relation to the elevated fasting insulin, the lipolysis and leptin signaling pathways in the sWAT were inhibited, favoring fat tissue deposition and consequent body fat gain, consistent with previous studies in which the insulin levels were genetically manipulated. However, this contradicted the CIM's predictions as there was a negative relationship between the fasting insulin and dietary carbohydrate content. Therefore, in relation to the decreased carbohydrate content rather than the increased carbohydrate content, the lipolysis and leptin signaling pathways in the sWAT were inhibited, contradicting the CIM's prediction. The hypothalamic hunger pathway was inhibited rather than stimulated in relation to the elevated fasting insulin, and the energy intake of the mice did not increase with the dietary carbohydrate. The daily energy expenditure of the mice was also not altered in relation to the carbohydrate content, although the browning pathway in the sWAT was downregulated in relation to the elevated fasting insulin (which demonstrated the decreased carbohydrate content). Therefore, there was no positive energy balance in the mice in relation to the elevated dietary carbohydrate content. In conclusion, the CIM did not explain the differences in the body fat of the mice in response to the altered dietary macronutrient composition in our study. It remains to be established if this is also true in humans, since the results may reflect a fundamental difference between mice and humans in the regulation of energy balance.

## Author contributions

S.H. was involved in the initial experiment design, conducted experiment one, analyzed the data from experiments one and two, performed the IPA-related analysis, and co-wrote the manuscript. L.W. was involved in the sample collection for experiments one and two and conducted the RNA extractions and the RNA-seq. J.T. was involved in the sample and data collection for experiments one and two. D.Y. and Y.X. performed the initial data collection and glucose measurements for experiment two. Y.W. conducted the insulin measurements and was involved in the initial data collection for experiment two. A.D. was involved in the RNA-seq-related analysis. J.R.S. directed both projects, conceived and designed the experiments, contributed to the data analysis, and co-wrote the paper. All of the authors approved the final version prior to submission for publication.
