# Supplementary material for: The carbohydrate-insulin model does not explain the impact of varying dietary macronutrients on the body weight and adiposity of mice
Source: Mol Metab. 2019 Nov 16;32:27–43. doi: 10.1016/j.molmet.2019.11.010 (PMC6938849; doi:10.1016/j.molmet.2019.11.010)
Supplement: Multimedia component 1 [file mmc1.pdf]

Supplemental figures:

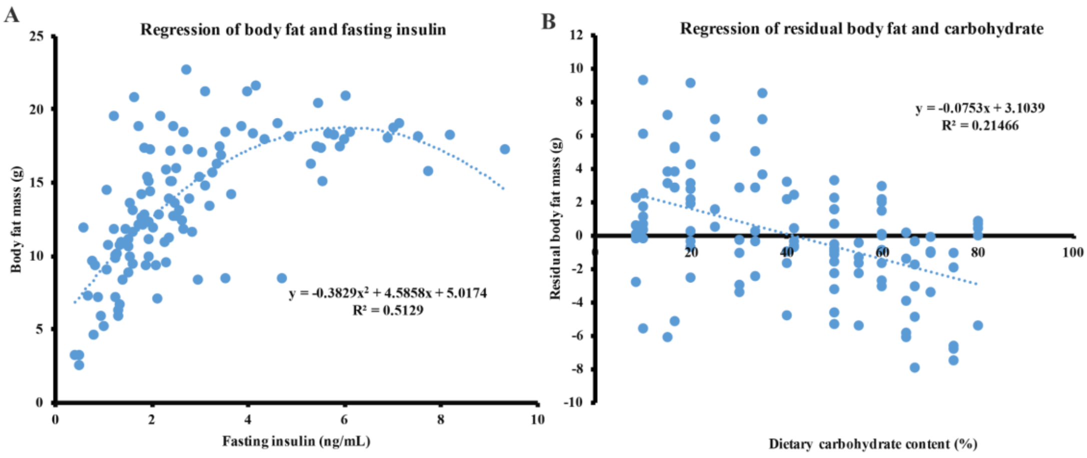

Figure S1 The regression between body fat mass and fasting insulin levels of the mice fed on experimental diets. Values were the measured values of each individual. (A) The regression between body fat mass and fasting insulin levels. (B) The regression between normalized body fat mass and dietary carbohydrate content. Related to Figure 1.

Supplemental table titles and legends:

Table S1 Genes from RNA sequencing of the sWAT in relation to fasting insulin levels. Related to Figure 2.

Table S2 Genes from RNA sequencing of the hypothalamus in relation to fasting insulin levels. Related to Figure 6.

Table S3 Composition of the 24 experimental diets. Related to STAR Methods.
